# Evaluating Extended Field of View Imaging for Measuring Rectal Tumor Lowest Boundary to Anal Verge Distance via Transrectal Biplane Ultrasound

**DOI:** 10.1055/a-2569-6939

**Published:** 2025-05-05

**Authors:** Yan Zhang, Lu Liang, Huachong Ma, Jiagang Han, Xiuzhang Lv, Huiyu Ge

**Affiliations:** 1 74639 Department of Ultrasound Medicine, Beijing Chaoyang Hospital Affiliated to Capital Medical University, Beijing, China; 2 74639 Radiology Department, Beijing Chaoyang Hospital Affiliated to Capital Medical University, Beijing, China; 3 74639 General Surgery Department, Beijing Chaoyang Hospital Affiliated to Capital Medical University, Beijing, China

**Keywords:** rectal cancer, colonoscopy, MR imaging, transrectal biplane ultrasound, extended field of view technology

## Abstract

**Purpose:**

This study aimed to measure the precise distance from the lowest boundary of a rectal tumor to the anal verge (DTAV) in patients with rectal cancer.

**Materials and Methods:**

A retrospective analysis was performed on clinical data from 70 rectal cancer patients. DTAV measurements were collected using transrectal biplane ultrasound, MRI, and colonoscopy.

**Results:**

The difference in DTAV measurements between the mean DTAV value obtained by ultrasound (US _mean_ ) and colonoscopy exhibited a difference of 0.22 cm. In contrast, the difference between US _mean_ and MRI was 0.48 cm, while the difference between MRI and colonoscopy was −0.26 cm. The ICC for DTAV measurements demonstrated excellent agreement, with values of 0.948 between US _mean_ and MRI, 0.942 between US _mean_ and colonoscopy, and 0.943 between MRI and colonoscopy. The minimum DTAV value obtained by ultrasound (US _min_ ) was 5.05 cm, the middle DTAV value obtained by ultrasound (US _mid_ ) was 5.10 cm, and the maximum DTAV value obtained by ultrasound (US _max_ ) was 5.30 cm. Notably, the median values of the differences in DTAV measurements between US _max_ and US _min_ , US _max_ and US _mid_ , as well as US _mid_ and US _min_ , were 0.2 cm, 0.1 cm, and 0.1 cm, respectively. Furthermore, the consistency of DTAV measurements between US _min_ and US _mid_ , US _max_ and US _mid_ , as well as US _min_ and US _max_ was excellent, with all ICC values reaching 0.999. Additionally, the radiologistʼs reassessment of MRI DTAV data showed excellent consistency with the original results, with an ICC value of 0.985.

**Conclusion:**

Transrectal biplane ultrasound utilizing EFOV imaging technology exhibited both accuracy and reproducibility for measuring DTAV. This approach provided a highly efficient and practical clinical tool for DTAV measurement.

## Introduction

The persistently high incidence and mortality rates of colorectal cancer remain a significant global health concern. According to authoritative statistical data 1 , the United States will experience a significant rise in the number of newly diagnosed colorectal cancer cases, reaching approximately 152,810 cases by 2024, thus making it the fourth most prevalent malignancy with the second highest mortality rate. Notably, rectal cancer comprises a substantial proportion of these cases, with an estimated 46,220 new cases annually, ranking second among newly diagnosed malignancies in the digestive system. Moreover, the incidence of rectal cancer has also been increasing rapidly in recent decades, particularly among younger individuals 2 . This trend poses a significant threat to individual health and quality of life, while also imposing a considerable burden on the social economy.

Despite significant advancements in treatment 3 , rectal tumors situated in various anatomical positions exhibit notable variations in treatment options, associated complications, pathological responses, and ultimate prognosis 4 5 6 . The disparities directly impact the quality of life of patients 7 . Among these considerations, precise measurement of the distance from the lowest boundary of the rectal tumor to the anal verge (DTAV) is a crucial factor in surgical planning 8 . The European Society for Medical Oncology (ESMO) classifies rectal cancer based on DTAV, distinguishing between low-position rectal cancer (≤5 cm), mid-position rectal cancer (>5–10 cm), and high-position rectal cancer (>10–15 cm) 9 . Typically, for early-stage rectal cancer with a DTAV less than 8 cm, transanal resection surgery is often the preferred surgical approach. For cT2~4N0~2M0 stage mid- to high-position rectal cancer, laparoscopic anterior resection surgery with annus preservation is routinely performed. Conversely, in the case of low-position rectal cancer, abdominoperineal rection surgery is often necessary due to the challenges associated with anus preservation 10 .

Despite the importance of determining DTAV, there is currently no definitive best method to accurately measure it. In clinical practice, the measurement of DTAV commonly involves methods such as digital rectal examination (DRE), magnetic resonance imaging (MRI), and colonoscopy. While DRE offers simplicity and convenience for initial assessment, its accuracy in detecting mid-to high-position rectal tumors is limited by the finger length of the examiner and the anatomical features of the patient. Additionally, the subjective nature of DRE assessments can introduce variability. MRI, a noninvasive and nonradioactive technology, plays a pivotal role in tumor localization and staging. However, there is currently no unified standard for positioning the anal verge and for measuring methods in this context 11 12 13 . Rigid sigmoidoscopy, on the other hand, provides a direct approach to examining and measuring tumor height, earning it the status of a gold standard for DTAV measurement. However, this method is associated with patient discomfort and limitations regarding insertion depth. In contrast, colonoscopy, with its flexible endoscope, has significantly improved patient comfort compared to sigmoidoscopy, gradually emerging as the preferred method for DTAV measurement 14 15 . Nevertheless, measurement errors may still occur if the endoscope is not aligned parallel to the rectal cavity. Therefore, it is crucial to ensure proper endoscope positioning and to consider these limitations when interpreting colonoscopy results for DTAV measurement.

With remarkable advancements in ultrasound technology, the introduction of innovative equipment, such as intracavitary end-fire probes, intracavitary biplane probes, and intracavitary 360° annular array probes, has underscored the pivotal role of ultrasound in rectal cancer diagnosis, staging, and therapeutic evaluation 16 . However, when it comes to measuring DTAV, the limited field of view of traditional ultrasound probes poses a challenge, with the comprehensive visualization of the sonographic image from the lowest boundary of the rectal tumor to the anal verge being impeded. Consequently, in practical scenarios, sonographers frequently need to combine multiple images to estimate the DTAV, which inevitably introduces measurement uncertainties. With the advancement of intracavitary biplane probes, ultrasound extended field of view (EFOV) imaging technology has emerged, leveraging the linear array mode of the intracavitary biplane probe. This technology incorporates advanced computer image processing techniques, enabling sonographers to continuously capture and process images through dynamic probe movements. By building upon traditional ultrasound imaging, EFOV imaging technology expands the imaging range, thereby facilitating comprehensive display of the tumorʼs involved length. This, in turn, establishes a robust foundation for precise DTAV measurement.

Previous research on EFOV imaging technology had primarily focused on measuring muscle length, area, and rectus abdominis curl angle 17 18 19 , and it was generally recognized as a reliable evaluation method for distance measurement beyond the length of the ultrasound probe. However, the application of EFOV imaging technology in rectal measurements, specifically for assessing the DTAV, remained uncharted territory. Consequently, this study aimed to explore the clinical application value of EFOV imaging technology within transrectal biplane ultrasound for measuring DTAV in rectal cancer.

## Materials and Methods

### Study Subjects

This study enrolled 84 patients who underwent rectal cancer staging diagnosis in the Ultrasound Medical Department from August 2022 to January 2024. Prior to the ultrasound examination, all patients had been definitively diagnosed with rectal cancer by means of colonoscopy and pathological examination. The exclusion criteria comprised: (1) 2 patients with tumors positioned too high for clear visualization due to the limited length of the intracavitary probe; (2) 4 patients with large tumors causing significant stenosis of the rectal lumen, impeding smooth insertion of the intracavitary probe into the rectal lumen; and (3) 8 patients who had not undergone MRI examination at our institution. As a result, the final study cohort comprised 70 patients with rectal cancer, ranging in age from 36 to 86 years, with a median age of 66 (IQR: 58, 77) years. The study employed a prospective data collection methodology, which was followed by a retrospective analysis, and approval for the study was obtained from the hospital ethics committee.

### Ultrasound Image Acquisition

The DTAV measurement was conducted using the EFOV imaging technology on the Mindray Resona 9 ultrasound system (Shenzhen Mindray Bio-Medical Electronics, Shenzhen, Guangdong Province, China), employing the ELC13–4U intracavitary biplane probes with a frequency range of 3.5–9.5 MHz for the convex array and 3.2–12.8 MHz for the linear array. Prior to the examination, patients underwent bowel cleansing via enema administration (110 mL of glycerol) administered through the rectum, one hour beforehand. During the procedure, the patient was positioned in the left lateral decubitus position, with hips and knees flexed, and the anus was fully exposed by separating the buttocks using the patientʼs right hand. The probe surface was coated with an adequate amount of coupling agent and subsequently covered with a latex sheath to guarantee optimal contact. Air was expelled from the sheath, and an additional layer of coupling agent was applied to its exterior.

The rectal ultrasound examination, utilizing the intracavitary biplane probe, was conducted by a seasoned sonographer with over two decades of expertise in transrectal ultrasonography. During the examination, dynamic grayscale images of the rectal tumor in both convex array and linear array modes were obtained. In the lithotomy position, the specific location of rectal tumors was marked and recorded using the clock face notation. In both scanning modes, the location of the tumorʼs lowest point was determined and verified independently. Upon confirmation of this location, the EFOV imaging function was activated in linear array probe mode by locating and clicking the panoramic imaging button on the ultrasound diagnostic equipmentʼs operating interface. During image acquisition, the probe was firmly pressed against the contralateral intestinal wall, with the surface of the linear array probe gently contacting the rectal wall on the side to be measured to ensure good coupling. Subsequently, following the preset direction and speed, the probe was smoothly and evenly moved from the tumorʼs upper edge to its lowest point and continued downward until the gas line at the anal edge was visible. Throughout this process, the real-time generation of the panoramic image on the screen was monitored to ensure completeness, clarity, and absence of significant distortion or artifacts. Based on the specific examination circumstances, parameters such as gain, time gain compensation, depth, and contrast were adjusted as necessary to achieve optimal panoramic image quality, clearly displaying the details and boundaries of tissues and organs.

**Fig. 1:**
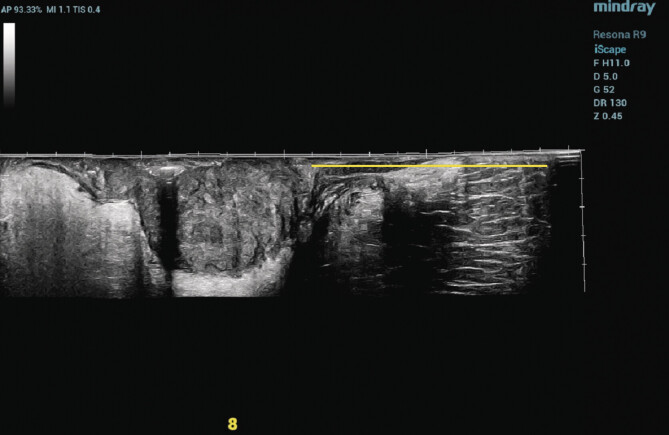
DTAV measurement is performed using EFOV imaging technology with an intracavitary biplane probe. The yellow line indicates the distance from the lowest boundary of the rectal tumor to the anal verge.

The entire measurement procedure was repeated three times, capturing grayscale images for each repetition. During each repetition, the distance DTAV was measured ( Fig. 1 ). After these measurements, the maximum DTAV value obtained by ultrasound (US _max_ ), the middle DTAV value obtained by ultrasound (US _mid_ ), and the minimum DTAV value obtained by ultrasound (US _min_ ) were recorded. The mean DTAV value obtained by ultrasound (US _mean_ ) was subsequently calculated based on these recorded measurements. It was important to note that the sonographer was blinded to the DTAV measurements derived from MRI and colonoscopy, ensuring the independence and reliability of the ultrasound-based measurements.

### MRI protocol and measurements

During rectal MRI examinations, the German Siemens Skyra 3.0T superconducting MRI scanner, equipped with an 18-channel body array coil, was deployed to guarantee exceptional image quality. Specifically, for imaging the sagittal plane, high-resolution turbo spin echo T2-weighted imaging (TSE-T2WI) technology was utilized. The scanning parameters were set as follows: repetition time (TR) 3200 ms, echo time (TE) 73 ms, slice thickness 3.0 mm, slice gap 10%, field of view (FOV) 150 mm x 150 mm, and a matrix optimized to 320 x 224 for enhanced image detail and clarity.

**Fig. 2:**
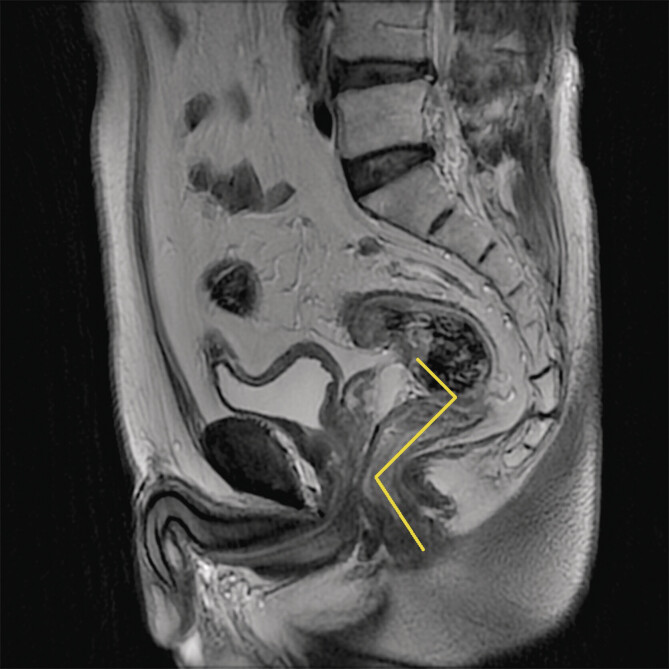
On sagittal T2-weighted MRI images, the DTAV is measured by tracing a yellow polyline along the rectal tract, and the cumulative length of this line is then used to determine the distance from the lowest margin of the rectal tumor to the anal verge.

An experienced radiologist with nine years of experience conducted a retrospective review of the pelvic MRI images stored in the picture archiving and communication system. The assessment focused on the precise measurement of DTAV, defined as the distance from the anal verge to the lowest boundary of the tumor, utilizing sagittal T2-weighted images. The radiologist traced the rectal pathway using polyline measurements from the anal verge to the tumorʼs inferior margin, adding the values to calculate the exact distance between these anatomical landmarks ( Fig. 2 ). It was important to emphasize that, during this process, the radiologist was unaware of the DTAV results derived from the ultrasound and colonoscopy measurements. Furthermore, the study gathered DTAV data from MRI reports authored by radiologists with varying degrees of experience, ensuring that the radiologist responsible for the current measurements remained blinded to the information contained in those reports.

### Colonoscopy Data Collection

The DTAV data, obtained through flexible colonoscopy, was meticulously gathered by thoroughly reviewing the medical records of the patients.

### Statistical Analysis

The collected data were systematically organized and comprehensively analyzed using SPSS 27.0 software. Accurate and informative charts and graphs were created with GraphPad Prism 9. For continuous variables exhibiting normal distribution, descriptive statistics were presented as mean±standard deviation. Conversely, for those displaying non-normal distribution, the median and interquartile ranges were used for characterization. Categorical variables were quantitatively represented by frequency (n) and percentage (%).

To assess the statistical significance of differences in DTAV measurements across technologies, the paired *t* -test was applied, with a *P* -value less than 0.05 indicating statistical significance. The consistency of continuous variables was rigorously evaluated using the intraclass correlation coefficient (ICC). ICC values≥0.75 indicated exceptional consistency, 0.4 to 0.75 suggested moderate to good consistency, and values<0.4 indicated poor consistency. Furthermore, scatter plots were constructed to visually represent the correlation between data points, thereby enhancing the interpretability of the results.

## Results

### General Information

A thorough pathological analysis was conducted on 70 patients, either via colonoscopy biopsy or surgical resection. The diagnosis identified 69 cases of rectal adenocarcinoma and a single case of neuroendocrine carcinoma. Among these patients, 29 underwent surgical intervention, 29 received neoadjuvant therapy, and 12 were assessed for their response to neoadjuvant therapy ( Table 1 ).

**Table:** **Table 1** General patient information.

*Variable*	*Values*
AGE ( *years* )	66 (58, 71)
Sex	
Male	46 (65.7%)
Female	24 (34.3%)
US _mean_ DTAV (cm)	5.17 (3.29, 7.20)
MRI DTAV (cm)	4.85 (2.88, 6.63)
Colonoscopy DTAV (cm)	5.00 (3.00, 7.00)
cT staging	
cT1	5 (7.1%)
cT2	7 (10%)
cT3	50 (71.4%)
cT4	8 (11.4%)
Pathology	
Adenocarcinoma	69 (98.6%)
Neuroendocrine carcinoma	1 (1.4%)
Measures	
Surgery	29 (41.4%)
NCRT	29 (41.4%)
After NCRT	12 (17.1%)
The lowest tumor point	
Anterior wall	37 (52.9%)
Posterior wall	33 (47.1%)

### A Comparative Analysis of DTAV Measurements Obtained Through Different Technologies

The analysis showed that the difference between US _mean_ and colonoscopy DTAV measurements was statistically insignificant, with a discrepancy of 0.22 cm ( *t* =1.999, *p* =0.050). Conversely, a significant statistical difference was observed between US _mean_ and MRI DTAV measurements, with a discrepancy of 0.48 cm ( *t* =4.621, *p* <0.001). Similarly, a significant statistical difference was observed between MRI and colonoscopy DTAV measurements, with a discrepancy of − 0.26 cm ( *t* =− 2.504, *p* =0.015). The results were summarized in Table 2 .

**Table:** **Table 2** A comparative analysis of discrepancies in DTAV measurements derived from three distinct methods.

Variable	MD	SD	*t*	*P*
*US*_*mean*_ *-Colonoscopy*	0.22	0.91	1.999	0.050
*US*_*mean*_ *-MRI*	0.48	0.87	4.621	< 0.001
*MRI-Colonoscopy*	− 0.26	0.87	− 2.504	0.015

MD: Differential value of DTAV measurements obtained from two examination methods.

### Consistency of DTAV Measurements across Diverse Methodologies

**Fig. 3:**
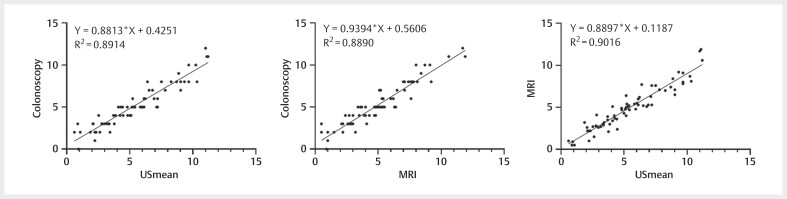
Scatter plots highlighted the favorable consistency in pairwise comparisons of DTAV measurements derived from three distinct methods.

**Fig. 4:**
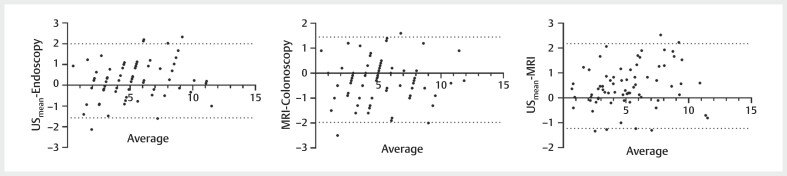
Bland-Altman plots highlighted the favorable consistency in pairwise comparisons of DTAV measurements derived from three distinct methods.

Fig. 3 presents scatter plots, and Fig. 4 presents Bland-Altman plots, both of which clearly illustrate the consistency among the three measurement technologies. Additionally, the ICC values presented in Table 3 indicate a high degree of agreement across all pairwise measurement methods.

**Table:** **Table 3** A comparative analysis of the consistency in pairwise DTAV measurements derived from three distinct methods.

*Variable*	ICC (95% CI)
US _mean_ and MRI	0.948 (0.917−0.967)
US _mean_ and Colonoscopy	0.942 (0.908−0.963)
MRI and Colonoscopy	0.943 (0.910−0.964)

### Assessment of the Reproducibility of Ultrasound-Based DTAV Measurements

The median DTAV values from ultrasound measurements were 5.05 cm (range: 3.25–7.03 cm) for the minimum (US _min_ ), 5.10 cm (range: 3.25–7.20 cm) for the middle (US _mid_ ), and 5.30 cm (range: 3.38–7.33 cm) for the maximum (US _max_ ). The median differences in DTAV measurements between US _max_ and US _min_ , US _mid_ and US _min_ , as well as US _max_ and US _mid_ were 0.2 cm, 0.1 cm, and 0.1 cm, respectively. Notably, the consistency between US _min_ and US _mid_ , US _min_ and US _max_ , as well as US _max_ and US _mid_ was excellent, with all ICC values reaching a value of 0.999 ( Table 4 ).

**Table:** **Table 4** Assessment of the reproducibility of ultrasound-based DTAV measurements.

*Variable*	ICC (95% CI)
US _min_ and US _mid_	0.999 (0.999−1.000)
US _min_ and US _max_	0.999 (0.998−0.999)
US _max_ and US _mid_	0.999 (0.999−1.000)

### Assessment of the Repeatability of MRI DTAV Measurements

MRI reported an average DTAV value of 5.0 cm (range: 3.00–7.00  cm). Upon re-measurement by the radiologist, the median DTAV value was 4.85 cm (range: 2.88–6.63 cm). The consistency between these two measurements was outstanding, attaining an ICC value of 0.985 (95%CI: 0.975–0.990).

## Discussion

This study marked the first application of EFOV imaging technology in transrectal biplane ultrasound for measuring DTAV in rectal cancer patients. The findings revealed that, when the transrectal biplane ultrasound linear array probe was employed, EFOV imaging technology not only fully visualized the entire length of the tumor but also offered a clear panoramic view that surpassed the physical limitations of the probe, encompassing the region extending from the lower margin of the tumor to the anal verge gas line. As a result, the precision and reliability of DTAV measurements were substantially enhanced.

To ensure the authenticity and reliability of this study, colonoscopy data were gathered by endoscopists with varying levels of experience, reflecting real-world clinical settings. The results showed that there was no statistically significant difference between US _mean_ and colonoscopy in DTAV measurements ( *t* =1.999, *p* =0.050), suggesting that ultrasound measurements were comparable to colonoscopy results. This minor discrepancy between the two methods was probably due to random error. However, a significant difference was observed between US _mean_ and MRI in measuring DTAV ( *t* =4.621, *p* <0.001), and similarly, a statistically significant difference was found between MRI and colonoscopy in DTAV measurements ( *t* =− 2.504, *p* =0.015). In this study, patients were positioned in the left lateral decubitus position for both ultrasound and colonoscopy examinations, whereas for MRI, they were scanned in the supine position. This difference in body positioning may have influenced the test results. Matthias et al. 20 conducted a study on tumor height measurement using rigid rectoscopy. They found good consistency between two researchers when measuring tumor height in the left lateral decubitus position. However, consistency was only moderate when measuring in the lithotomy position.

Jacobs et al. 21 conducted a study involving 211 patients and found that although MRI demonstrated good agreement with colonoscopy when measuring DTAV (ICC=0.7, 95% CI: 0.7–0.8), the values obtained were significantly smaller than those obtained by colonoscopy, with a difference of 2.5 cm (95% CI: 2.1–2.8). Similarly, Basendowah et al. 14 also found good consistency between MRI and colonoscopy when measuring DTAV (ICC=0.89, 95% CI: 0.48–0.99), but MRI measurements were smaller by 1.52 cm compared to colonoscopy. The results of this current study also exhibited a similar trend, showing that MRI-measured DTAV values were smaller than those of colonoscopy, with a difference of − 0.26 cm. Furthermore, this study found that the average ultrasound measurements were higher than those of MRI, with a difference of 0.48 cm. The analysis indicated that the differences in DTAV measurements may have originated from the varying measurement methods employed. Specifically, flexible colonoscopy necessitated air insufflation into the colonic lumen prior to examination. The flexibility of the colonoscope may have resulted in an elongation of the actual path distance traversed during clinical manipulation, thereby leading to larger DTAV measurements. In contrast, MRI calculated DTAV by drawing polylines along the sagittal plane of the evacuated rectal cavity and adding up the lengths of these polylines. Given the typical curved folds of the rectum, there were subjective differences among radiologists when identifying the turning points of the polylines. Furthermore, measuring along the polyline of the rectal lumen underestimated the length of the curved lumen, ultimately leading to MRI measurements being lower than the actual DTAV values.

During image acquisition, the ultrasound probe remained in contact with the rectal wall while being slid from the lowest point of the tumor to the edge of the anus. Therefore, ultrasound DTAV measurements were based on the state of the flattened intestinal wall. Previous scholars 22 had used MRI T2 sagittal images to delineate along the contour of the intestinal wall to determine the height of the tumor, which shared some similarity with ultrasound measurement. Their study revealed that the average tumor height, as measured by endoscopy, was (5.9±2.9) cm, whereas the average tumor height, as measured by MRI images, was (6.2±3.0) cm. When measured along the flattened intestinal wall on MRI images, the average tumor height was found to be greater than that obtained through endoscopy. This conclusion was in agreement with the findings obtained through the ultrasound measurement method employed in this study.

This study also revealed that the ICC values for DTAV measurements between US _mean_ and colonoscopy, MRI and colonoscopy, and US _mean_ and MRI were 0.942 (95% CI: 0.908–0.963), 0.943 (95% CI: 0.910–0.964), and 0.948 (95% CI: 0.917–0.967), respectively. These findings underscored the remarkable consistency among the three methods when measuring DTAV, establishing them all as dependable instruments for this measurement. Additionally, the study by Serracantet al. 23 echoed this sentiment. Their research findings further emphasized the high degree of reliability associated with MRI, EUS, and colonoscopy in evaluating DTAV. Specifically, when evaluating DTAV, the ICC between intraoperative rigid rectoscopy (IRR) and MRI was 0.870 (95% CI: 0.757–0.931), between IRR and EUS was 0.981 (95% CI: 0.968–0.989), and between IRR and colonoscopy was 0.872 (95% CI: 0.770–0.928).

From an ethical standpoint, this study considered it unethical to conduct multiple examinations by various individuals on the same patient, as it would cause unnecessary pain and impose an additional burden on the patient. Consequently, in this study, each patient underwent three repeated measurements conducted solely by one experienced sonographer, with no comparisons made among measurements by different sonographers. The consistency among these measurements was excellent, achieving an ICC of 0.999 for each. These findings strongly support the reproducibility of transrectal biplane ultrasound in DTAV measurements. Additionally, our study evaluated the consistency between re-measured MRI DTAV data by a single radiologist and previous DTAV data reports. The results exhibited excellent agreement, with an ICC of 0.985 (95%CI: 0.975–0.990), indicating the high reproducibility of MRI in DTAV measurements. Given its cost-effectiveness, ease of operation, and superior resolution, we recommend that transrectal biplane ultrasound EFOV imaging technology should be considered the preferred and crucial method for measuring DTAV in patients with rectal cancer.

Several limitations were identified in the study. Firstly, the retrospective research design precluded the use of a single endoscopist for colonoscopy, potentially compromising the accuracy and reproducibility of colonoscopy outcomes. Secondly, clinicians in clinical practice may have exhibited biases in their choice of ultrasonography, particularly a tendency to avoid performing it on patients with high-position rectal cancer, which could have introduced biases into the research findings. Furthermore, the sample size was relatively modest, and the study lacked support from multicenter investigations. Additionally, when applying EFOV imaging technology, differences in the starting points and movement paths chosen by various ultrasound physicians could have introduced bias into the images. Therefore, future studies should consider expanding the sample size and initiating multicenter collaborations to enhance their credibility and reliability.

In conclusion, transrectal biplane ultrasound with EFOV imaging technology demonstrated remarkable accuracy and reproducibility when measuring DTAV. This technology not only addressed the previous lack of ultrasound-based methods for DTAV measurement but also introduced an efficient and practical new approach for clinical DTAV assessment. Consequently, this study concluded that transrectal biplane ultrasound with EFOV imaging technology possessed significant clinical application value and merited widespread promotion and adoption.
